# Hybrid treatment of multifocal lung malignancy by concomitant transbronchial microwave ablation with same-session lung resection and post-lung resection ablation

**DOI:** 10.1093/icvts/ivaf152

**Published:** 2025-06-27

**Authors:** Aliss T C Chang, Joyce W Y Chan, Ivan C H Siu, Rainbow W H Lau, Cheuk Man Chu, Tony S K Mok, Calvin S H Ng

**Affiliations:** Department of Surgery, Prince of Wales Hospital, The Chinese University of Hong Kong, Hong Kong, China; Department of Surgery, Prince of Wales Hospital, The Chinese University of Hong Kong, Hong Kong, China; Department of Surgery, Prince of Wales Hospital, The Chinese University of Hong Kong, Hong Kong, China; Department of Surgery, Prince of Wales Hospital, The Chinese University of Hong Kong, Hong Kong, China; Department of Imaging and Interventional Radiology, Prince of Wales Hospital, The Chinese University of Hong Kong, Hong Kong, China; Department of Clinical Oncology, Prince of Wales Hospital, The Chinese University of Hong Kong, Hong Kong, China; Department of Surgery, Prince of Wales Hospital, The Chinese University of Hong Kong, Hong Kong, China

**Keywords:** transbronchial microwave ablation, hybrid operating room, multifocal lung cancer, video-assisted thoracoscopic surgery

## Abstract

**OBJECTIVES:**

Transbronchial microwave ablation may have additional value when performed with the same-session lung resection or in patients with a history of lung resection(s). We present our institutional cohort to assess the feasibility and safety of transbronchial microwave ablation with the presence of lung resection.

**METHODS:**

From March 2019 to February 2024, 92 patients who underwent transbronchial microwave ablation with either a history of major lung resection(s) or same-session ablation with concomitant video-assisted thoracoscopic lung resection(s) were included in this study. Procedural details, safety outcomes and length of stay were retrospectively analysed.

**RESULTS:**

There were 103 episodes of transbronchial microwave ablation performed, and 142 lung lesions were ablated. The average size of nodules was 11.80 mm. Technical success was 100% with a mean minimum margin of 6 mm. Complications occurred in 23 procedures; the majority were CTCAE grade 1 complications (74%), which resolved shortly with observation, and the rest were grade 2 and 3 complications (13%), including one case of bronchopleural fistula and two cases of pneumothorax that required drainage. The average length of stay was 1.46 days. A total of 11 cases of same-session ablation with lung resection were performed. The average procedural time was 226 min, which is significantly shorter than the 27 cases of separate surgery and ablation during the same period (*P *= 0.012).

**CONCLUSIONS:**

Performing transbronchial microwave ablation utilizing electromagnetic navigation bronchoscopic guidance is feasible and safe in the background of lung resection. This technique can also be incorporated into a one-stop treatment with concomitant lung resection.

## GRAPHICAL ABSTRACT

**Figure:**
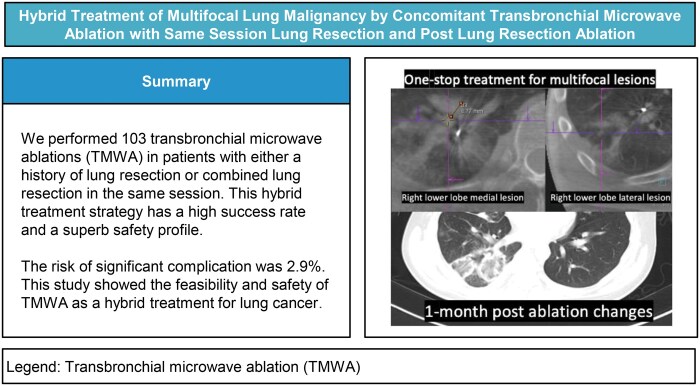

## INTRODUCTION

The incidence of multifocal, small and subsolid lung nodules is rising exponentially [1]. Despite their small size, these lung nodules should not be overlooked since they can harbour precancerous or early cancerous pathologies [2]. Both sublobar resection and local ablative therapy can be considered treatment modalities for these nodules with reasonable disease control [3], particularly when favourable features are seen on computed tomography (CT). Nevertheless, as surgical resection of multifocal lesions often results in sacrificing a significant amount of lung parenchyma and is associated with increased morbidity and mortality, various alternative local therapies have been studied in the hope of providing a hybrid multidisciplinary treatment that preserves as much pulmonary function as possible. Notably, stereotactic body radiation therapy (SBRT) and thermal ablation with either radiofrequency ablation or microwave ablation are the most frequently utilized modalities with a satisfactory loco-regional disease control rate [4–6]. Transbronchial MWA (TMWA) is a novel technique that has emerged as an alternate approach to local lung ablation, which the transbronchial route may allow better access to all areas of lung. Furthermore, previous studies showed an excellent technical success rate and a lower risk of pleural-based complications, even when ablating subpleural nodules [7–9]. TMWA can also be used concomitantly in multiple lung nodules to shorten total operative time and reduce the need for repeat general anaesthesia [10]. However, data on utilizing TMWA in patients with concomitant lung resection or following prior lung resection as a staged procedure is limited, and the safety profile in this setting needs to be addressed.

## PATIENTS AND METHODS

This is a single-centre retrospective analysis of patients who had either (i) a history of prior major lung resection for lung cancer and subsequently underwent TMWA or (ii) undergone concomitant lung resection with TMWA in the same session. Patients included were either diagnosed with biopsy-proven malignant nodules or radiologically suspicious nodules. All cases have been discussed in the thoracic multidisciplinary team (MDT) for evaluation for TMWA, including the presence of surgeons, medical and radiation oncologists, pulmonologists, radiologists, and pathologists. Radiological images and clinical background were reviewed in MDT in patients without a definite pathological diagnosis. Indications of surgery usually include suspicious radiological and clinical features, including nodules larger than 6 mm in size, nodules persisted and/or interval enlarged on serial scans, increased solid component on serial scans, and a prior history of cancer with nodules clinically compatible with multifocal lung cancer or metastasis. The feasibility of transbronchial ablation was evaluated by radiological features, such as the presence of bronchus sign, peripheral location and maximum size <3 cm are favourable. In case of suspected nodal involvement, nodal staging with endobronchial ultrasound was done to rule out nodal metastasis. This study was approved by the Joint Chinese University of Hong Kong—New Territories East Cluster Clinical Research Ethics Committee (CREC reference number: 2021.385). Consent of patients have been waived.

**Figure 1:**
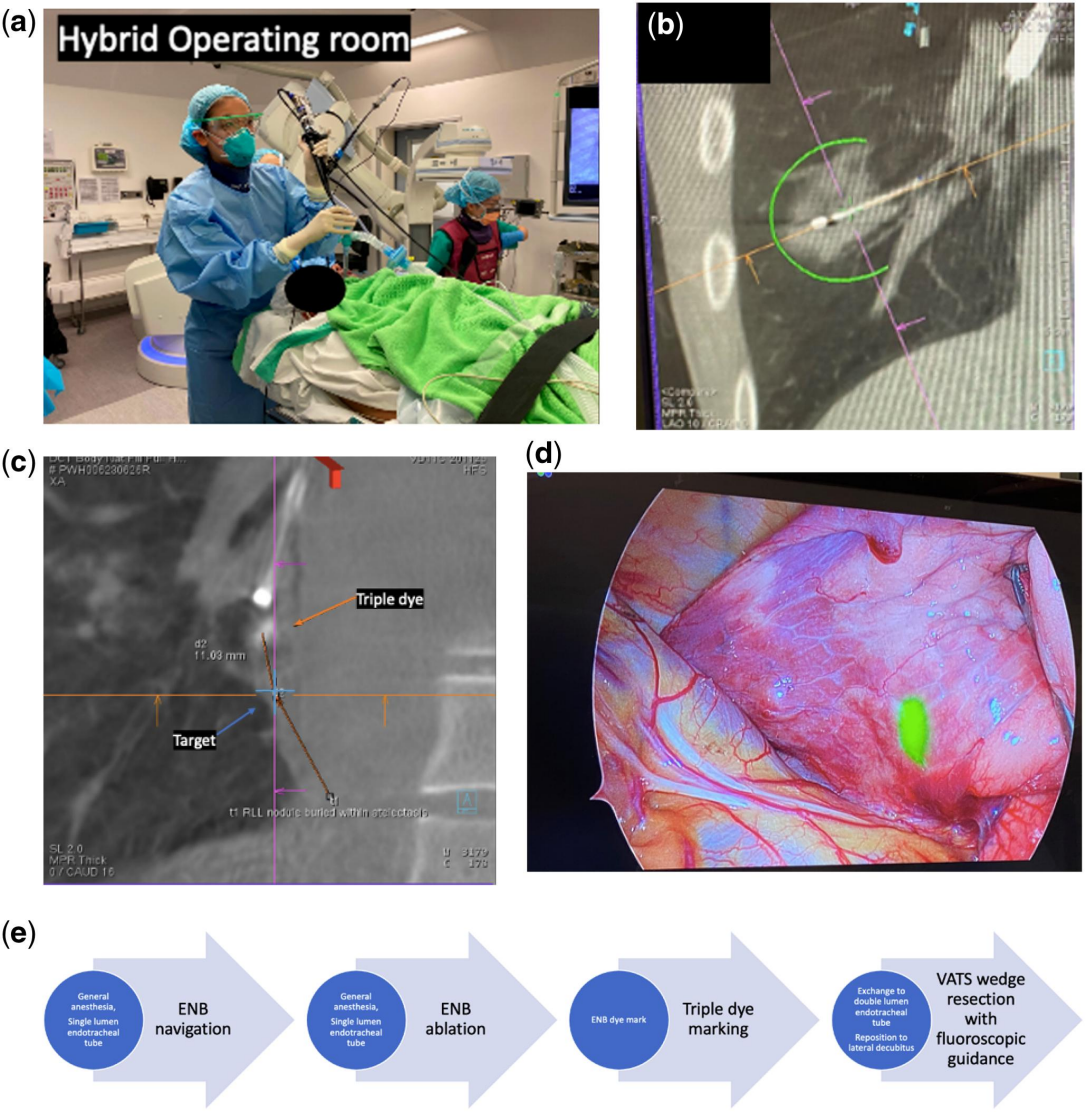
(**a**) HOR setup for bronchoscopic navigation under ENB guidance. (**b**) Real-time CBCT performed after TMWA to assess ablation zone coverage. (**c**) Real-time fluoroscopic image showing successful triple dye marking with clearly visualized hyperdense contrast agent near the target nodule. (**d**) Intraoperative near-infrared camera helps identify the Indocyanine green dye marking for accurate localization of nodule during VATS lung resection. (**e**) Schematic diagram of the routine workflow for a case of concomitant TMWA with ipsilateral wedge resection of the lung

Pre-operative thoracic CT was performed for transbronchial route planning. The hybrid operating room (HOR) is equipped with fluoroscopy, cone-beam CT (CBCT) and electromagnetic navigation bronchoscopy (ENB). The patient underwent general anaesthesia with a single-lumen endotracheal tube (Fig. 1a). Pre-navigation CBCT was performed after intubation to provide the most updated information. Afterwards, ENB navigation was done with either the SuperDimension or Illumisite Navigational Systems (Covidien, Plymouth, MN, USA). A locatable guide within an extended working channel was advanced towards the desired segmental airway. The locatable guide was exchanged for a CrossCountry or a fine needle aspiration needle, which was then deployed within the nodule. On-table use of CBCT and fluoroscopy allows fine adjustment or re-navigation if the needle tip placement is undesirable. After confirming the needle position, the ablation catheter was inserted. Another CBCT was used to confirm the position of the ablation catheter. TMWA was then performed to achieve a predicted ablation zone that provides complete coverage of the nodule and a circumferential minimal margin of at least 5 mm (Emprint ablation system, Medtronic Inc). A 10-min post-ablation CBCT was performed to assess the adequacy of the ablation zone and ensure sufficient margin (Fig. 1b). In case of under-ablation with inadequate margin or suboptimal coverage, re-ablation by adding more microwave energy might be performed by either: (i) re-ablation at the same position, (ii) pull-back the catheter for re-ablation, or (iii) re-navigation for bracket ablation [8, 11]. For patients who underwent concomitant lung resection, the single-lumen endotracheal tube was exchanged for a double-lumen one. The patient was then repositioned in preparation for video-assisted thoracoscopic surgery (VATS) (Fig. 1c–e).

Clinical assessment and chest radiography were performed daily throughout the in-hospital stay. The first postoperative outpatient follow-up was conducted 2 weeks after discharge. During the follow-up, chest radiography and a clinical assessment were performed. Afterwards, patients were closely followed up for up to 3 months postoperatively, and all patients received follow-up CT of the thorax for assessment on the post-ablation changes at 1 and 3 months following the procedure, then 6-month CTs thereafter with annual PET-CT scans (Supplementary Fig. S1).

All statistical analyses were done using Microsoft Excel and PRISM version 9.5.0. Continuous variables were presented as the mean, median and standard deviation. Categorical variables were presented as crude count and frequency (%). Student's *t*-test was used to compare the procedural time between groups, and we assumed the data was normally distributed and the observations were sampled independently. *P* values of ≤0.05 were considered statistically significant. Descriptive statistics were used for patient and nodule characteristics. The sample size was not estimated based on the study’s retrospective nature.

## RESULTS

From March 2019 to February 2024, 219 cases of TMWA were performed in the HOR. Of these cases, 92 patients were included in this cohort. There were 103 procedures performed and 162 lung nodules treated (142 lung nodules were ablated and 20 lung nodules were resected).

Patient characteristics are summarized in Table 1. The mean age was 62.8, ranging from 33 to 85. Reasons for ablation included confirmed or suspected multifocal lung malignancy (33.7%), history of major lung resection (22.8%), proven oligometastatic disease (20.7%), significant comorbidities or frailty (18.5%) and inadequate pulmonary function as defined as <40% predicted postoperative FEV1 and DLCO after the proposed lung resection (4.3%).

**Table 1:** Patient characteristics

		No. (%)	Mean	Range
Total no. of patient		92 (100)		
Age			62.8	33–85
Sex	Male	32 (34.8)		
	Female	60 (65.2)		
Main reason for ablation	Multifocal disease	31 (33.7)		
	History of major lung resection that makes reoperation unfeasible	21 (22.8)		
	Proven metastasis	19 (20.7)		
	Significant comorbidities/frailty	17 (18.5)		
	Inadequate pulmonary function	4 (4.3)		
History of major lung resection(s)		82 (89.1)		
Concomitant same-session lung resection		10^a^ (10.9)		
Smoking status	Active smoker	2 (2.2)		
	Ex-smoker	18 (19.6)		
	Non-smoker	72 (78.2)		
			Mean	SD
Pre-operative FEV1			90.4	17.2
Pre-operative DLCO			78.7	15.7

a One patient underwent separate sessions of concomitant ablation with lung resection.

Lung nodule characteristics are summarized in Table 2. The mean maximal diameter of the lung nodules ablated was 11.8 mm. Most of the ablated lung nodules were pure ground-glass nodules (47.9%); the rest were solid nodules (31.7%) and mixed ground-glass nodules with solid components (20.4%) [12]. Twenty-nine out of the ablated 142 lung nodules (20.4%) had pre-ablation biopsy performed, and 3 nodules were biopsied intra-operatively. The presence of bronchus sign occurred in 27 nodules (19%). The mean nearest distance from the pleura was 10.2 ± 9.2 mm.

**Table 2:** Nodule characteristics

		No. (%)
Total no of nodule ablated		142 (100)
Morphology [12]	GGO (Suzuki class 1–3)	68 (47.9)
	Mixed (Suzuki class 4–5)	29 (20.4)
	Solid (Suzuki class 6)	45 (31.7)
Multi-ablations to one nodule		94 (66.2)
No. of ablation to one nodule	Single	48 (33.8)
	Double	76 (53.5)
	Triple	14 (9.9)
	4 times	3 (2.1)
	5 times	1 (0.7)
	Mean	SD
Size (mm)	11.8	4.96
Minimum margin	6.02	2.61

The procedural characteristics are summarized in Table 3. The technical success rate of ablation was 100% in all 142 nodules with a mean minimal margin of 6 mm. Multiablation energy application to a single nodule occurred in 94 nodules (66.2%). The mean procedural time was 168.1 ± 81.4 min.

**Table 3:** Procedure characteristics

	No. (%)	Mean	SD
Total no. of procedure	103 (100)		
Concomitant ablations to multiple nodules in same-session	31 (30.1)		
Mean no. of nodules ablated in one session		1.38	0.66
Mean procedural time (min)		168.1	81.4
Mean procedural time in concomitant ablation with lung resection (min)		226.4	91.3
Mean procedural time in ablation with history of lung resection (min)		161.2	77.8

In the same-session ablation with lung resection group, 14 nodules were ablated and 20 were resected surgically. Eight cases had lung resection on the ipsilateral side. Three cases had concomitant ENB dye marking to aid intraoperative localization. In patients with concomitant ablation with ipsilateral lung resection, the mean nodule size was 8.2 ± 2.7 mm and the mean minimal distance from the nearest pleura was 16.9 ± 8.3 mm (Supplementary Table S1).

During the same study period, 27 patients underwent separate lung resection and ablation for multiple lung lesions, for which we extracted the data for comparison. The mean total procedural time for the separate lung resection and lung ablation sessions was 314 min. The mean total procedural time for the same-session ablation with lung resection was 226 min, which was significantly shorter (*P *= 0.012) compared to the separate sessions (Table 4).

**Table 4:** Comparison between concomitant ablation with same-session lung resection versus separate session of lung resection and ablation

	Concomitant ablation with same-session resection	Separate sessions of lung resection and ablation	*P*-value
No. of cases	11^a^	27	
No. of multiablation	8	17	0.575
Procedural time (min)	226	314	0.012

a Eleven sessions of concomitant ablation with lung resection were performed in 10 patients.

The average length of hospital stay in our cohort was 1.46 days. All patients had post-procedural follow-up CT at 3 months to monitor the presence of post-procedural complications; no periprocedural mortality or CTCAE grade 4/5 complication was reported, and 23 cases of postoperative complications were reported. Seventeen of which were CTCAE grade 1 complications which resolved without additional treatment. Three cases of grade 2 complications were reported, and they required further medical treatment, such as the use of oral antibiotics. Three cases of grade 3 complications were reported, including two cases of pneumothorax that required chest tube drainage and one case of bronchopleural fistula that required endobronchial valve placement on postoperative day 9 (Table 5). Two cases of complications required re-admission on postoperative day 7 and day 14 due to persistent fever and delayed pneumothorax, respectively. From our cohort, all complications presented themselves within a month after the procedure. We reported the loco-regional disease control using CT imaging and m-RECIST criteria. No early local recurrence was reported at 3 months. With a median follow-up time of 611.5 days, a total of four cases (2.82%) of local ablation site recurrence and three cases (2.11%) of lobar recurrence without ablation site recurrence were noted. Complete responses were achieved in 88 nodules (62.0%), partial responses were achieved in 35 nodules (24.7%) and stable disease was noted in 9 nodules (6.3%). There were 10 cases of progressive disease, and all of them had new or enlarging lung lesions in other parts of the lung.

**Table 5:** Safety outcomes and length of stay

	No. (%)	Mean	SD
Mean length of stay (LOS) (days)		1.46	1.24
LOS in concomitant lung resection with ablation		3.1	2.9
Total no. of complication	23 (22.3)		
CTCAE 1	17 (74)		
Pleuritic chest pain	6 (5.8)		
Haemoptysis	3 (2.9)		
Self-resolved fever	2 (1.9)		
Retention of urine	2 (1.9)		
Pleural effusion (not required drainage)	2 (1.9)		
Pneumothorax (not required drainage)	1(0.97)		
Rib fracture	1 (0.97)		
CTCAE 2	3 (13)		
Persisted fever	1 (0.97)		
Airway bleeding	2 (1.9)		
CTCAE 3	3 (13)		
Pneumothorax required drainage	2 (1.9)		
Bronchopleural fistula	1 (0.97)		
CTCAE 4/5	0 (0)		

## DISCUSSION

The discovery of multifocal lung nodules through increasing utilization of CT is becoming more prevalent [13]. Even though guidelines suggested that the management of multiple lung nodules should be based on the most suspicious nodule [14], other studies indicated that treating additional suspicious nodules would affect the survival and prognosis [15]. Pretreatment tissue diagnosis for multifocal lung nodules is desirable but often problematic, as the diagnostic yield of percutaneous or conventional bronchoscopic biopsy is likely to be suboptimal due to the small size and the ground-glass nature [16]. In this study, 29 nodules (20.4%) had pretreatment histopathological confirmation, and only three cases underwent the same-session biopsy prior to ablation. This low number in pretreatment pathological confirmation is due to the technical difficulty in performing a biopsy in small nodules and due to the often small number of cells retrieved due to the ground-glass nature. Moreover, the limited use of same-session biopsy is due to our observation of local pulmonary haemorrhage at the biopsy site that creates ground-glass changes on CT. The nodules ablated in this study are generally small, and the majority were pure ground-glass nodules. Thus, even a minor degree of pulmonary haemorrhage from biopsy would obscure the nodule border and make ablation zone assessment difficult on CT scans. Hence, thoracic MDT is vital in management decision-making, especially when the pretreatment histological diagnosis cannot be obtained. Radiological and clinical features were carefully examined in the thoracic MDT. Indications for treatment are usually nodules with highly suspicious features, including enlarging lesions and/or an increase in solid component, often in the background of a previous history of proven multifocal lung cancer or lung metastases.

Surgery as the sole treatment for multiple lung nodules is associated with morbidity and significant loss of pulmonary function, which is often poorly tolerated. Thus, hybrid treatment with lung resection and TMWA would be a potential treatment option to minimize the lung volume loss. For instance, the Emprint ablation catheter can produce an ellipsoid ablation zone of 4.2 × 3.5 cm maximally. The estimated volume of ablated lung parenchyma of the maximum ablation zone is approximately 27 cm^3^, and this volume loss is undoubtedly less than a sublobar resection required for a lung nodule >1 cm. Such advantage of ablation over surgical resection is even more significant when the lung nodule is deep within the lobe. We have reported eight cases of ipsilateral lung resection ablation. The mean nodule size in these cases was 8.2 mm while the minimal distance from the nearest pleura was 16.9 mm, and the distance from pleura is relatively large compared to their size. If surgical resection alone were used in these cases, a significant lung volume would be sacrificed for a comparatively small nodule. Therefore, taking the nodule size and depth into account, small and deep nodules were treated with ablation, and we aim to treat all lung nodules in the same session while preserving as much healthy lung parenchyma as possible.

Despite the anatomical distortion after prior lung resection, our ENB navigation achieved a superb success rate without requiring special adjustments. The specialized software offered by the navigation system can automatically create optimal pathways by utilizing 3D reconstructed CT, allowing highly accurate bronchial mapping for TMWA in post-lung resection patients. Furthermore, only 19% of the ablated nodules had bronchus signs present during operative planning. With transbronchial access tools like the CrossCountry tool (Covidien, Plymouth, MN, USA), peripheral lung nodules with no bronchus sign can be reached. Hence, anatomical restriction is not a prominent issue in TMWA in our experience, and navigation to all lung segments is feasible.

Another advantage of performing TMWA with concomitant lung resection is the variety of intraoperative localization techniques that we can perform using the ENB system. We performed ENB triple dye marking (mixing a 1:1:1 ratio of Indocyanine Green, Methylene blue, and Iodohexol) for localization before lung resection [17, 18]. This mixed dye agent offers excellent visualization of the marking that can be seen by multiple imaging modalities. ENB localization enhanced the ease of resecting small, subsolid lung nodules and shortened procedural time by enabling a one-stop treatment in the HOR. The procedural time in concomitant ablation with VATS was significantly shorter than those who had separate sessions performed; this can be explained by the absence of transferal time between the operating room and the ward, and the avoidance of the repeat general anaesthesia.

This study addressed the feasibility of TMWA as part of a hybrid treatment strategy. In this study, a thoracic MDT assessment was required in all patients and TMWA was offered for nodules highly suspected of being malignant. The early to mid-term local control in this cohort is favourable, with no local recurrence detected on CT at 3 months. The median follow-up time in this cohort was 611.5 days, and the overall rate of local recurrence was approximately 5%, indicating nearly 95% of patients remained recurrence-free and over 86% of ablated nodules showed complete resolution or shrinkage. Compared to the reported local disease control rates of SBRT and percutaneous thermal ablation (i.e. freedom from local recurrence from 78% to 96% at 2- to 5-year follow-up) [4, 19], the local control in TMWA appears comparable. However, the lack of histopathological confirmation in some cases complicates the comparison and statistical interpretation of the treatment efficacy. The NAVABLATE study used the same technology for TMWA in 30 pathologically confirmed primary lung cancer and metastases. The technical success was 100%, and 1-month imaging showed 100% treatment efficacy [20]. Combining these promising results, we proposed the need for a larger prospective study to evaluate the long-term efficacy of TMWA.

In addition to the feasibility, the safety outcomes of performing a series of complex procedures are important. Among 103 procedures, only 23 cases of postoperative complications were reported, with the majority being minor complications. No procedure-related mortality or major complications (CTCAE 4 and 5) were reported up to 3 months after the procedure. From our experience gained from this cohort, post-procedural complications often presented within 1 month after the procedure.

SBRT is an alternative lung-preserving treatment, for which Choi *et al.* reported a local control rate calculated per lesion to be 88.2% at three years. However, they also reported 22.5% of acute or late toxicity in their cohort [21]. The key disadvantage of SBRT is the potential radiation toxicity, mainly when used in multiple nodules, multiple fractions with escalated doses, or when targeting central tumours [22, 23]. A study in 2021 found that multicourse radiation to multiple nodules is correlated to an increased incidence of radiation toxicity, particularly chest wall toxicity [24]. Hence, TMWA is a safer option with the freedom from radiation, especially in patients who are susceptible to radiation toxicity.

Percutaneous thermal ablation has an outstanding local control rate, but also has significant weaknesses, including anatomical restriction and pleural-based complications. Lung nodules shielded by bony prominence or centrally located are technically challenging to access percutaneously. Since the visceral pleura is constantly breached in percutaneous ablation, pleural-based complications such as pneumothorax are frequent [25, 26]. Moreover, heat energy conduction via the chest wall can lead to osteonecrosis and rib fractures to nearby ribs [27]. In contrast, the risk of pleural-based complications is lower in TMWA [26, 28, 29] because it avoids direct pleural puncture. From our observation, the ablated lung or pleura is toughened and unlikely to rupture. Nonetheless, pleural-based complications can still occur in the transbronchial route, from manipulating the instrument during navigation or advancing the access needle or ablation catheter. During the exchange of the instruments, due to opposing forces from the tumour, a sudden instrumental “give-way” could occur, resulting in potential puncture of the nearby pleura. Despite their rarity, we observed the occurrence of pneumothorax and bronchopleural fistula. Among the patients who developed sizeable pneumothorax that required drainage, pleural drainage is frequently the only necessary treatment. This is because pneumothorax usually arises from a pleural puncture during instrument manipulation, and the punctured site would typically heal with adequate drainage. We observed one bronchopleural fistula, which was demonstrated on postoperative CT. With radiological imaging confirming the diagnosis, this patient eventually underwent a bronchoscopic examination and received an endobronchial valve placement that provided immediate stoppage of air leak. The valve was removed 6 weeks following placement. Although bronchopleural fistula is uncommon, further intervention to prevent air leakage is essential. Additionally, the ablation catheter in the TMWA does not have a fixed anchor point, unlike the percutaneous approach. Hence, heat energy from the catheter is less likely to transfer back to the chest wall and cause thermal injury to the chest wall or intrapleural nerve endings.

This study shows TMWA has a safer profile than percutaneous thermal ablation or SBRT, avoiding radiation toxicity and reducing pleural complications, which occur in up to 52% of cases [26]. The overall complication rate was 22%, mostly minor and resolving with minimal or no treatment. While not completely risk-free, TMWA offers an excellent safety profile compared to other local ablative therapies, with fewer significant side effects and complications.

### Limitation

This study has several limitations, including its retrospective nature and small cohort size, which may affect the generalizability of findings. The low complication rate associated with TMWA indicates the need for a larger population to establish significant statistical differences and ensure adequate power. Additionally, only short to mid-term safety outcomes were evaluated, necessitating longer follow-ups for assessing long-term effects on oncological outcomes and treatment efficacy. The absence of histopathological diagnoses is a significant limitation, as it complicates the interpretation of oncological disease control efficacy. Despite thorough assessments in the MDT, the lack of definitive pathology may overestimate effectiveness. Future prospective studies are needed to better evaluate oncological outcomes, although current data are promising for using TMWA as a hybrid treatment.

## CONCLUSION

Given the favourable safety profile and high success rate in this study, TMWA in HOR is a safe and feasible option as a hybrid treatment when combined with VATS lung resection for multifocal lung nodules. In the era of multifocal disease, this hybrid approach could enhance our patient's prognostic outcomes while preserving pulmonary function to improve post-treatment quality of life.

## Supplementary Material

ivaf152_Supplementary_Data

## Data Availability

The data underlying this article cannot be shared publicly due to the privacy of individuals that participated in the study. The data will be shared on reasonable request to the corresponding author.

## ABBREVIATIONS

CBCT: Cone-beam computed tomography
CT: Computed tomography
ENB: Electromagnetic navigation bronchoscopy
HOR: Hybrid operating room
MDT: Multidisciplinary team
SBRT: Stereotactic body radiation therapy
TMWA: Transbronchial microwave ablation
VATS: Video-assisted thoracoscopic surgery
