# Supplementary material for: Hybrid treatment of multifocal lung malignancy by concomitant transbronchial microwave ablation with same-session lung resection and post-lung resection ablation
Source: Interdiscip Cardiovasc Thorac Surg. 2025 Jun 27;40(7):ivaf152. doi: 10.1093/icvts/ivaf152 (PMC12237502; doi:10.1093/icvts/ivaf152)
Supplement: ivaf152_Supplementary_Data [file ivaf152_supplementary_data.zip › Supplementary table 1.docx]

**Supplementary material**

Table 1. Operative details in patients received same-session transbronchial MWA and VATS lung resection

| **Patient no.** | **Ablated nodule location** | **Lung nodule ablated** | **Side of concomitant lung surgery** | **Operation performed** | **Lung nodules resected** | **Total nodules treated** | **Complications** | **CTCAE grade** | **Length of stay (days)** | **Final pathology of resected nodules** |
| --- | --- | --- | --- | --- | --- | --- | --- | --- | --- | --- |
| 1 | Right upper lobe | 1 | Ipsilateral | Uniportal VATS right middle lobe wedge resection | 1 | 2 |  | 0 | 2 | Hepatocellular metastasis |
| 2 | Right upper lobe | 1 | Contralateral | VATS Lingular wedge resection | 1 | 2 |  | 0 | 2 | Hepatocellular metastasis |
| 3 | Right lower lobe | 1 | Ipsilateral | ENB dye marking + VATS right lower lobe inferior and medial wedge resection | 1 | 2 |  | 0 | 2 | Leiomyosarcoma metastasis |
| 4 | Right lower lobe | 1 | Ipsilateral | VATS right upper lobectomy | 2 | 3 |  | 0 | 2 | 2 foci of primary lung adenocarcinoma |
| 5 | Right upper lobe | 1 | Ipsilateral | VATS right middle lobe wedge resection | 1 | 2 |  | 0 | 2 | Colorectal metastasis |
| 6 | Left upper lobe anterior + posterior segment | 2 | Ipsilateral | VATS left lower lobe apical segmentectomy | 1 | 3 | Bronchopleural fistula, required endobronchial valve placement | 3 | 11 | Primary adenocarcinoma of lung |
| 7 | Left upper lobe | 1 | Ipsilateral | ENB dye marking + VATS left lower lobe + left upper lobe wedge resection | 2 | 3 |  | 0 | 1 | 4 foci AIS / MIS / AAH |
| 8 | Right lower lobe lateral + medial segment | 2 | Ipsilateral | VATS wedge resection of right upper lobe x 2 + right middle lobe x 1 | 3 | 5 |  | 0 | 2 | Colorectal metastasis |
| 9 | Right upper lobe | 1 | Contralateral | ENB dye marking + VATS left upper lobe wedge resection | 1 | 2 | Pleuritic chest pain | 1 | 2 | Negative for malignancy |
| 10 | Left lower lobe posterior + anterior segment | 2 | Contralateral | VATS right middle lobe wedge x 2 + right lower lobe wedge x 4 | 6 | 8 |  | 0 | 6 | Parathyroid carcinoma metastasis |
| 11 | Right middle lobe | 1 | Ipsilateral | VATS right lower lobe wedge resection | 1 | 2 | Intra-operatively noted airway mucosal bleeding, treated with Adrenaline spray | 2 | 2 | 2 foci of primary lung adenocarcinoma |
