# Supplementary material for: Hybrid treatment of multifocal lung malignancy by concomitant transbronchial microwave ablation with same-session lung resection and post-lung resection ablation
Source: Interdiscip Cardiovasc Thorac Surg. 2025 Jun 27;40(7):ivaf152. doi: 10.1093/icvts/ivaf152 (PMC12237502; doi:10.1093/icvts/ivaf152)

Supplementary figure 1. CT images of 1-month and 3-months post-ablation changes on a right lower lobe ablation zone.


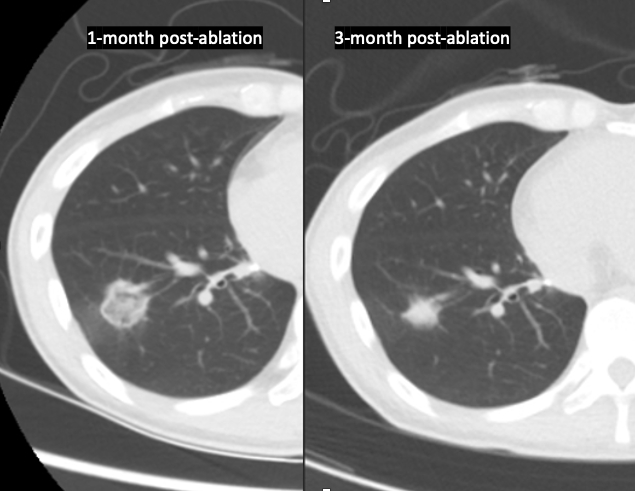

Supplement: ivaf152_Supplementary_Data [file ivaf152_supplementary_data.zip › Supplementary figure 1.docx]
